# Use of a modified early warning score system to reduce the rate of in-hospital cardiac arrest

**DOI:** 10.1186/s40560-016-0134-7

**Published:** 2016-02-09

**Authors:** Isao Nishijima, Shouhei Oyadomari, Shuuto Maedomari, Risa Toma, Chisato Igei, Shinya Kobata, Jyun Koyama, Ryuichiro Tomori, Natsuki Kawamitsu, Yoshiki Yamamoto, Masafumi Tsuchida, Yoshihiro Tokeshi, Ryo Ikemura, Kazufumi Miyagi, Koichi Okiyama, Kiyoshi Iha

**Affiliations:** Department of Critical Care Medicine, Chubu Tokushukai Hospital, 3-20-1, Teruya, Okinawa City, Okinawa Japan; Department of Internal Medicine, Chubu Tokushukai Hospital, Okinawa, Japan; Department of Surgery, Chubu Tokushukai Hospital, Okinawa, Japan; Department of Neurosurgery, Chubu Tokushukai Hospital, Okinawa, Japan

## Abstract

**Background:**

Physiological abnormalities are often observed in patients prior to cardiac arrest. A modified early warning score (MEWS) system was introduced, which aims to detect early abnormalities by grading vital signs, and the present study investigated its usefulness.

**Methods:**

Based on previous reports, the Chubu Tokushukai Hospital-customized MEWS was developed in Okinawa, Japan. The MEWS was calculated among all inpatients, and the rates of in-hospital cardiac arrests (IHCAs) were compared according to the score. The warning zone (WZ) was set as 7 or more because of the high possibility of acute deterioration. The MEWS system was introduced to provide immediate interventions for patients who reached the WZ in accordance with the callout algorithm. The numbers of IHCAs were compared between the 18 months before and after introduction of the MEWS system.

**Results:**

The numbers of patients who experienced IHCA with each score were as follows: score of 6, 1 of 556 patients (0.18 %); score of 7, 4 of 289 (1.40 %); score of 8, 2 of 114 (1.75 %); and score of 9 or more, 2 of 56 (3.57 %). There was no significant difference in the mean age or sex between before and after the introduction of the MEWS system. The rate of IHCAs per 1000 admissions decreased significantly from 5.21 (79/15,170) to 2.05 (43/17,961) (*p* < 0.01).

**Conclusions:**

The Chubu Tokushukai Hospital-customized MEWS was applied to all inpatients, and the rate of IHCA decreased owing to the introduction of the system, as the system enables early interventions for patients who have the possibility of acute deterioration.

## Background

Patients who experience cardiac arrest or who are in need of intensive care unit (ICU) management often show signs of deterioration in clinical findings several hours prior to the event [1–5]. It is possible that avoidable mortality occurs when these signs are missed and appropriate treatment is not provided [6, 7]. The original early warning score (EWS) system was designed to enable early detection of patient abnormalities using major vital signs prior to deterioration into a critical illness [8]. The modified early warning score (MEWS) system, which uses modified physiological parameters for scoring, has proven to be a useful tool for predicting deterioration in patients. The MEWS is a guide for medical staff to better recognize patients’ conditions prior to deterioration and to enable them to provide early interventions [9–11]. Furthermore, since vital signs are used in the system for detection, the proficiency of the medical staff does not affect the results. Drower et al. reported that the incidence of cardiac arrests per 1000 admissions significantly decreased from 4.67 in 2009–2010 to 2.91 in 2010–2011 after the introduction of the MEWS system at a 600-bed tertiary teaching hospital in New Zealand [12]. However, evidence proving a decrease in in-hospital cardiac arrests (IHCAs) because of the introduction of the MEWS system is limited. The system was introduced to decrease the occurrence of IHCA, and its usefulness was studied by comparing evaluations of IHCA before and after the introduction of the system.

## Methods

Chubu Tokushukai Hospital in Okinawa, Japan, is an acute care hospital with 331 beds, and the major departments are as follows: internal medicine, cardiology, surgery, cardiovascular surgery, pediatrics, neurosurgery, urology, and orthopedics. In addition, the major inpatient diseases include pneumonia, angina, and urinary tract infection. Before the introduction of the MEWS system at the hospital, when patients showed signs of deterioration, the ward nurses judged its extent and contacted an attending physician; however, there was no standardized protocol for nurses to use as criteria for judgment. Moreover, when an IHCA occurred, a hospital-wide announcement was made, and regardless of the department, all available physicians rushed to the ward to perform cardiopulmonary resuscitation. The MEWS system was introduced with the intention to provide a system for the early detection of patients who present with acute deterioration before the occurrence of IHCA.

Table 1 shows the hospital scoring of the MEWS. This scoring was referred to in the MEWS system modified by Gardner-Thorpe et al., which enables early screening for patients who need intensive care because of surgical disease [13]. A MEWS modified by Subbe et al. is applicable to acute internal disease and indicates an increase in mortality risk if the score is high [11]. Blood pressure, pulse rate, respiratory rate, body temperature, consciousness, and any concern about a patient’s condition were each given a score of 0–3, and the sum of the scores was calculated. A higher score indicates increased severity. Although urine volume per hour is included in the items of the MEWS modified by Garner-Thorpe et al. [13], our hospital excluded it because our target included all inpatients at the hospital, and this parameter is difficult to measure in all patients. Moreover, the item regarding “any concern about the patient’s condition,” which is considered a key factor when demanding support in the activation criteria of the medical emergency team (MET) [14], was added to our hospital’s MEWS system. The MEWS system was used routinely on all inpatients. An evaluation was conducted one or more times each day depending on the patient’s illness severity, and the highest score was targeted in this study. A system was introduced to calculate the MEWS automatically when vital signs were entered into the patient’s medical record by a ward nurse.

**Table 1:** The modified early warning score (MEWS) system

Score	3	2	1	0	1	2	3
Systolic blood pressure (mmHg)	≤70	71–80	81–100	101–199		≥200	
Heart rate (bpm)		≤40	41–50	51–100	101–110	111–129	≥130
Respiratory rate (bpm)		≤8		9–14	15–20	21–29	≥30
Temperature (°C)		≤35.0		35.1–38.4		≥38.5	
Conscious level				Alert	Reacting to voice	Reacting to pain	Unresponsive
Any concern about the patient’s condition				No	Yes		

The MEWS is calculated by summing the parameters one or more times a day, depending on the severity of the patient’s condition

The specific score was defined as the warning zone (WZ), since a higher score is associated with a greater possibility of acute deterioration. In order to set a proper WZ, the number of IHCAs according to the score over a 7-month period between October 1, 2012, and April 30, 2013, was discussed. Table 2 shows the numbers of patients and IHCAs according to the score. The numbers of patients who experienced an IHCA with each score were as follows: a score of 6, 1 of 556 patients (0.18 %); a score of 7, 4 of 289 (1.40 %); a score of 8, 2 of 114 (1.75 %); and a score of 9 or more, 2 of 56 (3.57 %). There was a significantly higher IHCA rate among patients with scores of 7, 8, and 9 or more than among patients with scores of 6. If the WZ is set at a score of 6 or more, the number of false negatives would increase; therefore, a score of 7 or more was considered an appropriate setting for the WZ.

**Table 2:**
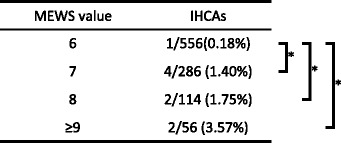
The number and frequency of in-hospital cardiac arrests (IHCAs) according to the modified early warning score (MEWS)

The frequencies of IHCAs were as follows: score of 6, 0.18 %; score of 7, 1.40 %; score of 8, 1.75 %, and a score of 9 or more, 3.57 %. The frequency of IHCAs was significantly higher among patients with scores of 7 or more than among patients with scores of 6

* p < 0.05

**Fig. 1:**
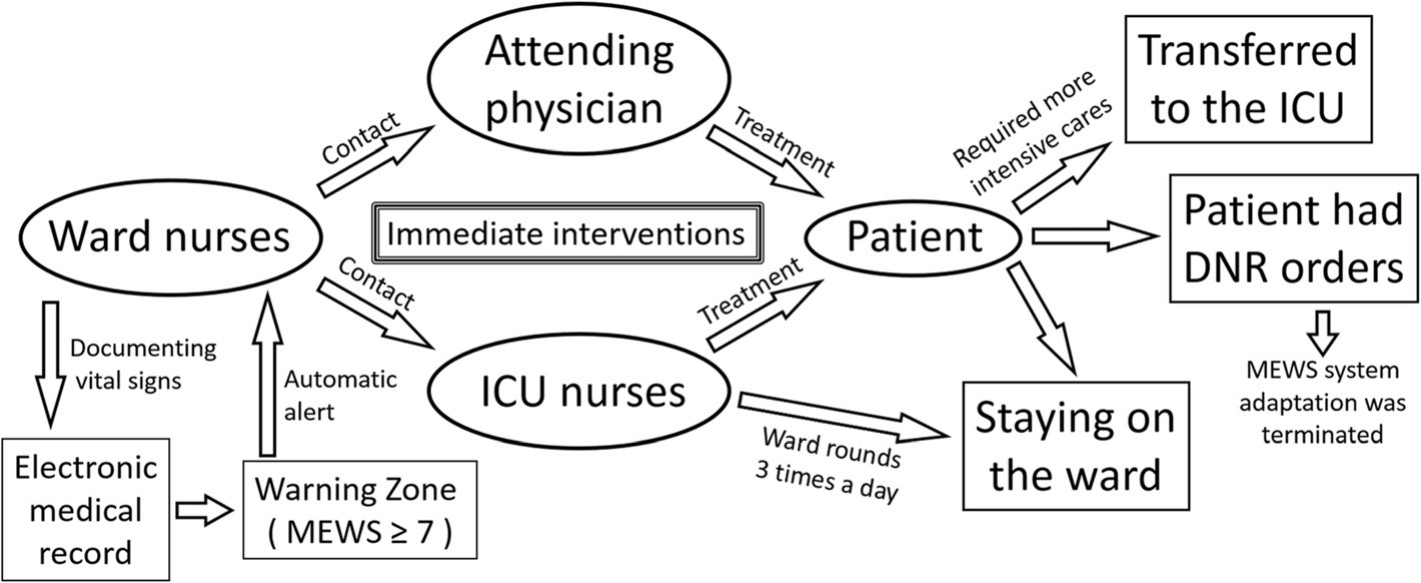
Callout algorithm. The modified early warning score (MEWS) is automatically calculated when documenting vital signs in the electronic medical record, and a warning alarm is activated if the score reaches the warning zone. After receiving a warning, the ward nurses contact an attending physician and intensive care unit (ICU) nurses immediately. Patients receive initial and continued treatment in the ward and are transferred to the ICU. If the patient has do-not-resuscitate (DNR) orders, the MEWS system adaptation is terminated

The MEWS system was introduced to enable immediate interventions for patients who reached the WZ in accordance with the callout algorithm (Fig. 1). When the MEWS reached the WZ, an automatic alert was generated in the electronic medical record to inform the ward nurses to contact an attending physician and the intensive care nurses to provide the required initial management to improve the patient’s condition in the ward, including arranging a transfusion, administering a vasopressor, and arranging for an artificial respirator. In cases where the patient’s condition continued to deteriorate after the initial response, and for those who required more intensive care, the patient was transferred to the ICU. However, if the patient had do-not-resuscitate (DNR) orders, the MEWS system adaptation was terminated. ICU nurses continually monitored the scores of all inpatients through their electronic medical records, and inpatients with severe conditions who were present in the ward were treated during ward rounds three times a day.

The numbers of IHCAs during each 18-month period before the introduction of the MEWS system (from April 1, 2011, to September 30, 2012) and after the introduction of the MEWS system with a WZ setting of 7 or more (from October 1, 2013, to March 31, 2015) were compared. Patients in need of ICU management and patients with DNR orders were excluded. The primary end point was set as an IHCA, and statistical significance was defined as *p* < 0.05, using Fisher’s exact test and the chi-squared test for validation. All statistical analyses were performed using SPSS Statistics 22 (IBM Corp., Armonk, NY, USA). This study was reviewed and approved by the ethics committee of Chubu Tokushukai Hospital.

## Results

Table 3 shows the patients’ backgrounds before and after the introduction of the MEWS system. No significant differences were noted between the two periods (15,462 inpatients vs. 17,961 inpatients; mean age, 58.5 ± 29 years vs. 59.3 ± 28 years (*p* > 0.05); males, 51.4 vs. 54.4 % (*p* > 0.05)). The top three diagnoses prompting hospital admission were pneumonia, angina, and urinary tract infection.

**Table 3:** Patients’ backgrounds

		Before the introduction of the MEWS system		After the introduction of the MEWS system	
Admissions			15,170		17,961
Age, mean ± SD			58.5 ± 29		59.3 ± 28
Male, *n* (%)			7790 (51.4 %)		9762 (54.4 %)
Diagnoses prompting hospital admission, *n* (%)	1	Pneumonia	922 (6.1 %)	Pneumonia	1160 (6.5 %)
	2	Angina	582 (3.8 %)	Angina	797 (4.4 %)
	3	Urinary tract infection	399 (2.5 %)	Urinary tract infection	439 (2.4 %)
	4	Acute gastroenteritis	355 (2.3 %)	Congestive heart failure	386 (2.1 %)
	5	Congestive heart failure	340 (2.2 %)	Acute gastroenteritis	368 (2.0 %)

Comparisons of the number of admissions, age, sex, and diagnosis prompting the admission during periods before and after the introduction of the modified early warning score system are shown. There were no significant differences in age or sex, and the top diagnoses prompting hospital admission were roughly identical

As Table 4 shows, during the study period, there were 122 IHCAs in total. The number of patients who reached the WZ was 920, and the monthly mean was 51.1. Furthermore, the number of in-hospital deaths was 550 before introduction of the MEWS system and 636 after introduction of the MEWS system. The in-hospital mortality rate per 1000 admissions was not significantly different (36.3 vs. 35.4; *p* > 0.05).

**Table 4:** The number of patients who reached the warning zone (WZ) and the rate of in-hospital cardiac arrests (IHCAs)

	WZ	IHCAs	Admissions	Incidence of IHCAs per 1000 admissions	In-hospital deaths
Apr 2011		6	804	7.46	31
May 2011		2	814	2.46	39
Jun 2011		4	855	4.68	27
Jul 2011		4	854	4.68	22
Aug 2011		14	831	16.85	31
Sep 2011		7	792	8.84	41
Oct 2011		5	868	5.76	28
Nov 2011		3	818	3.67	24
Dec 2011		3	820	3.66	28
Jan 2012		3	843	3.56	26
Feb 2012		4	769	5.20	35
Mar 2012		7	868	8.06	36
Apr 2012		3	827	3.63	31
May 2012		1	839	1.19	19
Jun 2012		1	827	1.21	37
Jul 2012		4	965	4.15	34
Aug 2012		5	911	5.49	26
Sep 2012		3	865	3.47	35
Oct 2013	29	2	940	2.13	37
Nov 2013	39	0	994	0.00	34
Dec 2013	44	2	956	2.09	35
Jan 2014	101	6	1016	5.91	61
Feb 2014	62	1	876	1.14	31
Mar 2014	65	1	1005	1.00	31
Apr 2014	48	4	1088	3.68	31
May 2014	50	1	1057	0.95	40
Jun 2014	34	3	1029	2.92	42
Jul 2014	84	4	1125	3.56	36
Aug 2014	28	1	986	1.01	32
Sep 2014	60	2	987	2.03	37
Oct 2014	36	2	965	2.07	29
Nov 2014	33	3	894	3.36	35
Dec 2014	55	3	1033	2.90	31
Jan 2015	72	4	1013	3.95	28
Feb 2015	46	3	950	3.16	34
Mar 2015	34	1	1047	0.96	32

The number of inpatients during each 18-month period before and after the introduction of the modified early warning score system. The monthly mean number of patients who reached the WZ was 51.1

**Fig. 2:**
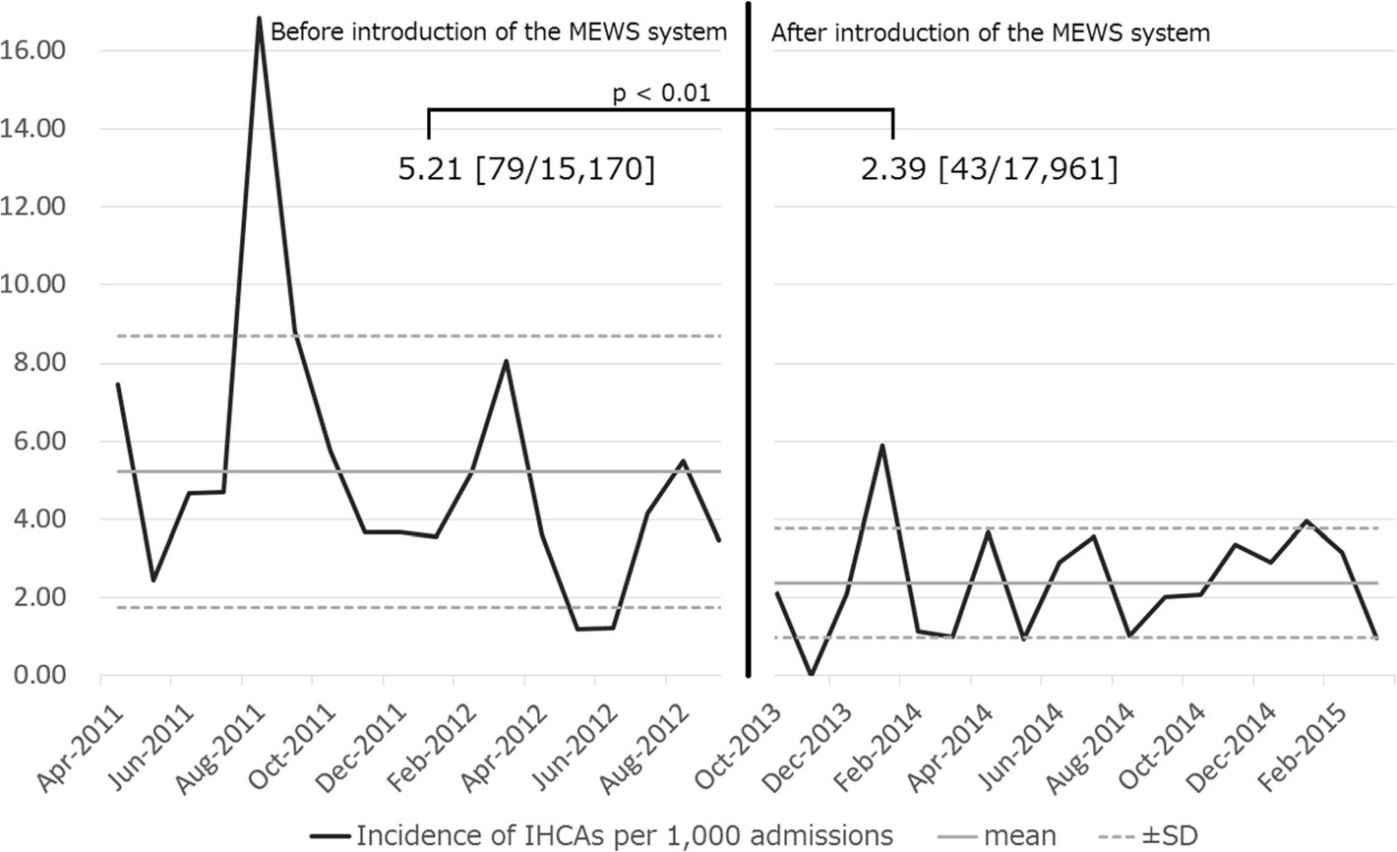
The monthly in-hospital cardiac arrest (IHCA) incidence. The *straight line* in the middle is the mean, and the *dashed lines* at the top and bottom of the figure indicate ±1 standard deviation (SD). The incidence of IHCAs per 1000 admissions is significantly lower after introduction of the modified early warning score system than before introduction of the system

Figure 2 shows the monthly incidence of IHCAs per 1000 admissions. The straight line in the middle is the mean, and the dashed lines at the top and bottom of the figure indicate ±1 standard deviation. A significant decrease in the number of IHCAs was noted (before the introduction of the MEWS system, 5.21 (79/15,170); after introduction, 2.39 (43/17,961); *p* < 0.01).

## Discussion

Clinical assessment tools based on vital signs have been developed to screen for signs of disease deterioration at an early stage. The EWS system, which was first reported by Morgan et al. in 1997, is a clinical assessment tool that scores systolic blood pressure, pulse, respiratory rate, and body temperature and if the patient is alert, responds to voice, responds to pain, or is unresponsive (AVPU) [9]. The MEWS includes a revised form of the item for blood pressure, and it is changed from normal blood pressure to urine volume per hour, which has led to the identification of surgical patients who can benefit from intensive care [10]. Subbe et al. later adapted this for internal medicine patients, and statistical tests showed that low body temperature was present in the most serious cases [11]. From the results of the score revision, it was concluded that the system was also useful for internal medicine patients. In emergency cases, the patient’s previous normal blood pressure data is often unavailable and lacks simplicity; accordingly, our hospital used measured values. Furthermore, an important factor for requesting support to start a MET was “feeling worried about the clinical condition” [14]; thus, we added “any concern about the patient’s condition” to the MEWS system in our hospital.

In 1995, Lee et al. introduced a MET in Liverpool Hospital in Australia to provide early screening for and treatment of patients at risk for cardiac arrest [15]. In 1999, Goldhill et al. formed the Patient at Risk Team to respond to patients in the hospital ward who presented with physiological abnormalities, and the number of cases of cardiopulmonary arrest decreased significantly from 30.4 to 3.6 % [16]. In 2001, Buist et al. performed an analysis of MET efficacy and reported that the number of cases of unexpected cardiac arrest decreased by 50 % and that the mortality rate decreased from 77 to 55 % [17].

It is possible that under normal circumstances, the MET should handle WZ patients. However, because of the lack of human resources to form a MET at our hospital, the initial response is made by an attending physician and nurses in the ICU. The introduction of the MEWS system has significantly decreased the number of IHCAs from 5.21 to 2.39 per 1000 admissions. It was reported that in 358 American hospitals, the total number of IHCA cases was 102,153, and the number of IHCA cases per 1000 admissions was 4.02 [18]; thus, introducing a MEWS system was considered to be effective. In order to evaluate all inpatients using a MEWS system, the previously reported MEWS system was modified. Moreover, it is considered that the decrease in the number of IHCAs resulted from setting the WZ and implementing our hospital’s original system to provide an immediate response for patients with the possibility of acute deterioration. In Japan, Taniguchi presented general remarks of the rapid response system (RRS) and MET in 2014 [19]. However, there are no reports of the effects of an introduced RRS or MET and RRS using a MEWS system. This study is the first report from Japan that showed the influence of the RRS using a MEWS system on clinical outcomes.

A limitation of this study is that conducting comparisons among patient populations before and after the introduction of a MEWS system is difficult. There were no significant differences in age, sex, the top three diagnoses prompting hospital admission, and the number of in-hospital deaths; however, it is difficult to exclude other factors that are considered to contribute to a decrease in the number of IHCAs. Furthermore, “any concern about the patient’s condition” was added to the MEWS system at our hospital and given a score of 1; however, a future evaluation is required to verify the appropriateness of the score. This report is based on a small number of cases from a single institution, and its statistical strength is weak. For these reasons, further studies on the MEWS system are necessary at multiple institutions or with a randomized design covering adaptable and non-adaptable groups.

In 2012 in England, a national EWS that included supplementary oxygen administration and percutaneous oxygen saturation scores was proposed and unveiled [20]. We expect that various tools for clinical assessment will be proposed in the future.

## Conclusions

By introducing a MEWS system and setting the WZ to 7 or more, the attending physician and ICU nurses could provide initial treatment to patients immediately, which led to a significant decrease in the incidence of IHCAs.
